# Dynamic local connectivity uncovers altered brain synchrony during propofol sedation

**DOI:** 10.1038/s41598-017-08135-2

**Published:** 2017-08-17

**Authors:** Rose Dawn Bharath, Rajanikant Panda, Jitender Saini, Kamath Sriganesh, G. S. Umamaheswara Rao

**Affiliations:** 1 Department of Neuroimaging and Interventional Radiology, National Institute of Mental Health and Neurosciences (NIMHANS), Bangalore, India; 2 Department of Neuroanaesthesia and Neurocritical Care, National Institute of Mental Health and Neuroscience (NIMHANS), Bangalore, India

## Abstract

Human consciousness is considered a result of the synchronous “humming” of multiple dynamic networks. We performed a dynamic functional connectivity analysis using resting state functional magnetic resonance imaging (rsfMRI) in 14 patients before and during a propofol infusion to characterize the sedation-induced alterations in consciousness. A sliding 36-second window was used to derive 59 time points of whole brain integrated local connectivity measurements. Significant changes in the connectivity strength (Z Corr) at various time points were used to measure the connectivity fluctuations during awake and sedated states. Compared with the awake state, sedation was associated with reduced cortical connectivity fluctuations in several areas connected to the default mode network and around the perirolandic cortex with a significantly decreased correlation of connectivity between their anatomical homologues. In addition, sedation was associated with increased connectivity fluctuations in the frequency range of 0.027 to 0.063 Hz in several deep nuclear regions, including the cerebellum, thalamus, basal ganglia and insula. These findings advance our understanding of sedation-induced altered consciousness by visualizing the altered dynamics in several cortical and subcortical regions and support the concept of defining consciousness as a dynamic and integrated network.

## Introduction

Human consciousness is an enigma, and states of reversible loss of consciousness are useful channels to unravel this mystery. Understanding the neurobiological basis of sedation has, thus, captured the interest of many scholars, and studies using resting state fMRI (rsfMRI) have provided important but contradictory evidence of increased^1–3^, decreased^4–6^ or heterogenous connectivity^1, 7^. The discrepancy in the results could be partially due to the varying analysis techniques, and the majority of studies have used hypothesis driven techniques, such as region of interest (ROI) and seed to voxel analyses^2, 4, 6, 8^, that focus on a default mode network (DMN) and a sensorimotor network; some studies have used data driven global (independent component analysis (ICA))^7^, local (integrated local connectivity (ILC))^5^ and intrinsic connectivity contrast (ICC)^1^ connectivity measures. The levels of sedation are also known to alter functional connectivity, and lower sedation levels (Observer Assessment of Alertness/Sedation scores (OAAS) 3 and above) are linked to an increased somatosensory cortical connectivity^2, 3, 7^, while deeper levels of sedation (OAAS 3 and below) are linked to a decrease cortical connectivity^1, 5, 9^. Several multistage studies exploring awake, sedated, unconscious and recovery states have captured the dynamic changes in connectivity during the loss and recovery of consciousness^10, 11^. One of the major limitations of these connectivity methods is that the measurement is averaged over the data acquisition duration and, hence, does not consider the fluctuations or variability in the system.

According to a large scale dynamic model of consciousness^12^, human consciousness is not static and exists at the verge of instability between a multi-stable attractor state and an unstable spontaneous state^13, 14^. This state of instability is considered essential as it assumes that the system is periodically monitoring and is maximally sensitive to any external stimulus^15^. Experimental investigations of consciousness also reveal a dynamic state of connectivity between several networks^13, 16^, particularly those involving the parietal and frontal cortices^16^. Dynamic connectivity (DC) rsfMRI analysis^17^ is a novel technique that uses sliding temporal windows to yield continuous series of connectivity snap shots that can be used to characterize the temporal evolution of networks. This technique has been used extensively in rsfMRI in both humans and animals^18–23^ and  has been applied to characterize disease states, such as epilepsy^24^, Alzheimer’s disease^25^, schizophrenia^26^ and task performance^18, 27^.

There is increasing evidence that the information obtained using static and dynamic methods is different, and static connectivity measures are more dependent on the structural anatomy and dynamic connectivity (DC), which is a phenomenon that is independent of the brain structure^15^. A recent DC study exploring propofol-induced loss of consciousness in rats reported a substantial reduction in the temporal variance in brain connectivity during sedation^28^. Another dynamic connectivity analysis using a spatial co-activation pattern (CAP), which measures connectivity dynamics during supra-threshold time periods, has also found reduced dynamics during sedation^29^.

Based on this dynamic concept of connectivity, we re-analysed previously acquired data^9^ during awake and sedated states in patients with low backache undergoing imaging under IV propofol sedation using DC. Our hypothesis stated that the sedation-state will have significantly reduced connectivity fluctuations compared to the awake-state based on evidence from previous studies. To measure the dynamic local connectivity (DLC), a 36 second time window was established to “slide” through the entire data, and a Fisher’s Z transformed ILC map was derived for each window. These maps were compared between conditions and averaged across windows to derive areas that revealed significant changes with sedation. Beta values, which are a measure of the connectivity strength, were calculated from each sliding window, and their fluctuations^30^ were measured. The between-region correlation and multiband frequency analysis were also performed to further characterize the temporal covariance and frequency specificity of these fluctuations. This study has enabled us to capture the dynamics of the resting brain under sedation and used fMRI to validate theoretical models of consciousness.

## Results

In total, 14 patients (M:F, 8:6; mean age 46.9 ± 11.3 years) successfully completed both the baseline awake and sedated state fMRI imaging. No patient required an alteration in the dose of the propofol infusion (1.5 mg/kg/hour) during the fMRI acquisition to achieve and maintain the desired clinical end-point-modified Observer Assessment of Alertness/Sedation (OAAS) score of 3 to 2. The changes in the cardiovascular (heart rate and mean BP) and respiratory parameters (SpO_2_ and ETCO_2_) during the imaging period (baseline and sedated state) were not significantly different (p > 0.05) according to the repeated-measures ANOVA analysis, minimizing the possible effect of these parameters on the BOLD signal changes.

**Figure 1:**
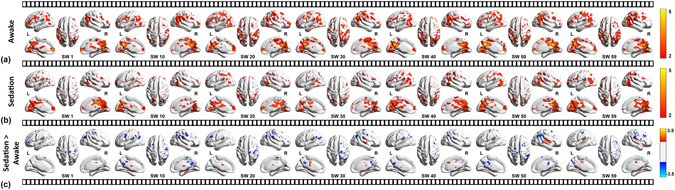
Dynamic functional connectivity during the awake state, sedation and the differences between two conditions is depicted in rows. Seven representative snap shots of integrated local correlation at sliding window 1, 10, 20, 30, 40, 50 and 59 is shown overlapped on BrainNet viewer images. Decreased cortical connectivity is represented in blue and increased deep nuclear connectivity is depicted in red in the last row which represents the effects of sedation.

Compared with the baseline, the sedated state was characterized by a diffuse decreased cortical connectivity along with increased deep nuclear DLC fluctuations as demonstrated in Fig. 1.

### Dynamic Local connectivity analysis

**Figure 2:**
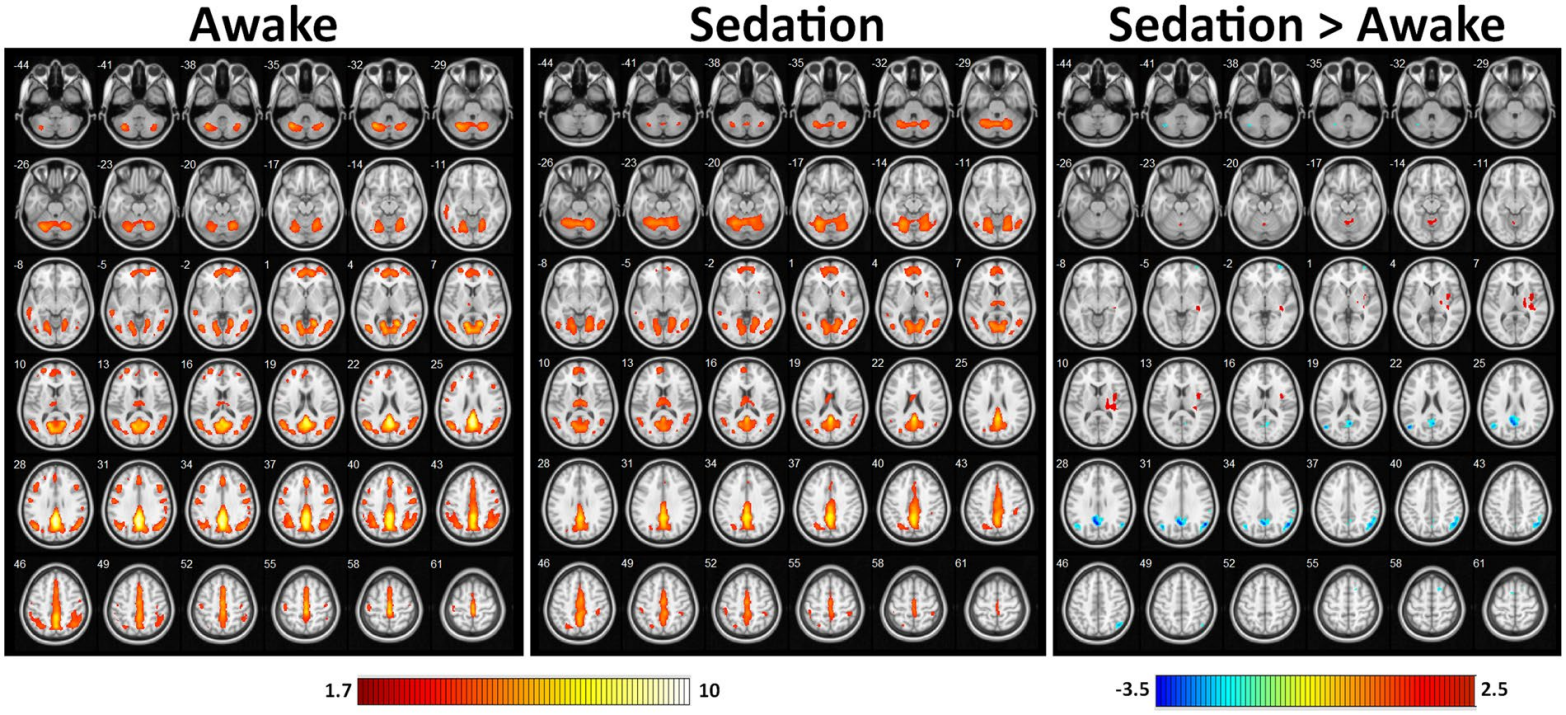
Whole brain group mean image of dynamic functional connectivity maps during the awake state, sedation and the differences between two conditions. The regions which showed significant decreased connectivity prominently involving the default mode network and peri-rolandic regions is represented in blue and the regions with increased connectivity involving cerebellum, thalamus and basal ganglia is depicted in red in the last column which depicts effects of sedation.

Among all the voxels evaluated, the regions that revealed the most significant (FDR P < 0.05) connectivity changes during sedation are shown in Fig. 2 and Table 1. The cortical regions included components of the DMN (posterior cingulate cortex {PCC}, pre-cuneus, angular and left middle temporal gyrus), perirolandic regions (right pre- and post-central and bilateral supplementary motor cortex), right middle frontal gyrus, right supra-marginal gyrus, and crus 1 and 2 of the left cerebellum. In addition, the cerebellar (bilateral cerebellum 4 and 5 and vermis 4 and 5), right diencephalic (thalamus) and basal ganglionic (putamen and insula) structures revealed increased connections during sedation.

**Table 1:** Regions revealing significant DLC differences between awake and sedation state.

No	X (coor)	Y (coor)	Z (coor)	Brain Region	ILC-Connectivity	Cluster Size	T - Value	P-Value
1	−2	−60	30	Precuneus	Decrease	457	3.3	0.003
2	1	−51	25	Posterior Cingulate Cortex	Decrease	178	2.4	0.006
3	52	−62	32	Right Angular	Decrease	422	3.1	0.005
4	57	−41	42	Right Supra Marginal Gyrus	Decrease	42	1.9	0.045
5	49	−10	37	Right Post-central Gyrus	Decrease	43	2.2	0.03
6	50	−12	40	Right Pre-central Gyrus	Decrease	69	2.3	0.026
7	16	16	60	Right Supplementary Motor Area	Decrease	13	1.4	0.05
8	−4	6	62	Left Supplementary Motor Area	Decrease	16	1.8	0.047
9	34	62	−2	Right Middle Frontal	Decrease	18	2.1	0.03
10	−48	−68	22	Left Middle Temporal Gyrus	Decrease	55	2.9	0.014
11	34	−5	8	Right Putamen	Increase	58	−2.5	0.012
12	38	0	10	Right Insula	Increase	147	−2.5	0.011
13	20	−16	8	Right Thalamus	Increase	85	−2.4	0.022
14	−6	−56	−14	Left Cerebellum_4_5	Increase	44	−2.4	0.022
15	8	−54	−14	Right Cerebellum_4_5	Increase	24	−2.4	0.032
16	−2	−58	−12	Vermis_4_5	Increase	57	−2.3	0.022
17	−38	−66	−40	Left Cerebellum Crus 1 & 2	Decrease	58	2.2	0.03

The t-values represent the strength of ILC connectivity between the voxel and its neighboring correlated voxels. Cluster size represents the number of voxels correlated in the brain region. The p-value represents the corrected significance level of the ILC connectivity.

**Figure 3:**
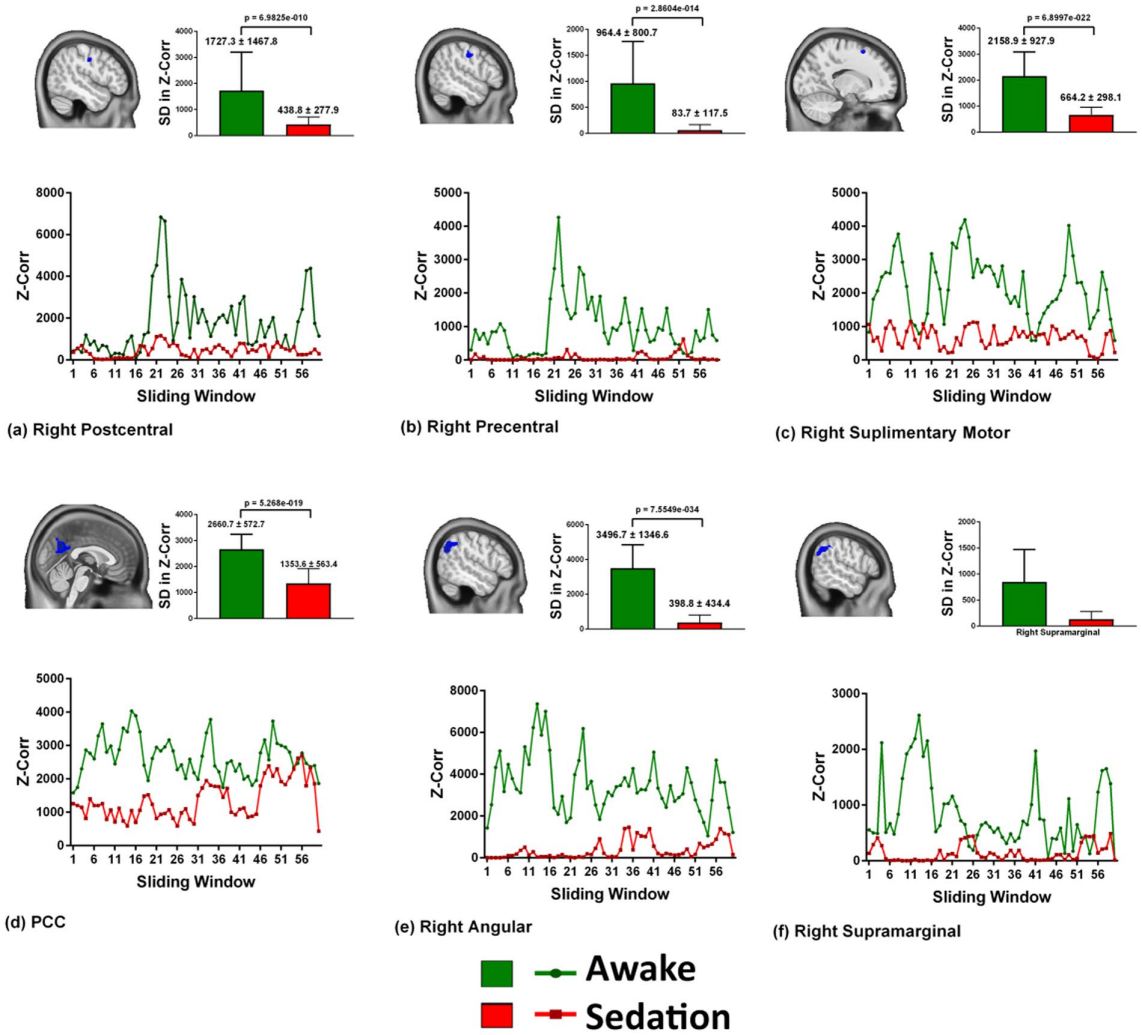
Connectivity fluctuations. Six representative cortical regions which revealed decreased dynamic functional connectivity (DLC) during sedation is presented. The strength of the Z correlation on the Y-axis is plotted across the sliding window time points from 1 to 59 on the X-axis. The DLC at baseline is depicted in green and during sedation is shown in red. The bar graph in the inset reveals the standard deviations (SD) in the Z correlation strengths during the two conditions. Significant sedation-induced decrease in connectivity of the cortical regions involving the default mode network and the peri-rolandic regions with decreased SD indicating decreased fluctuations.

**Figure 4:**
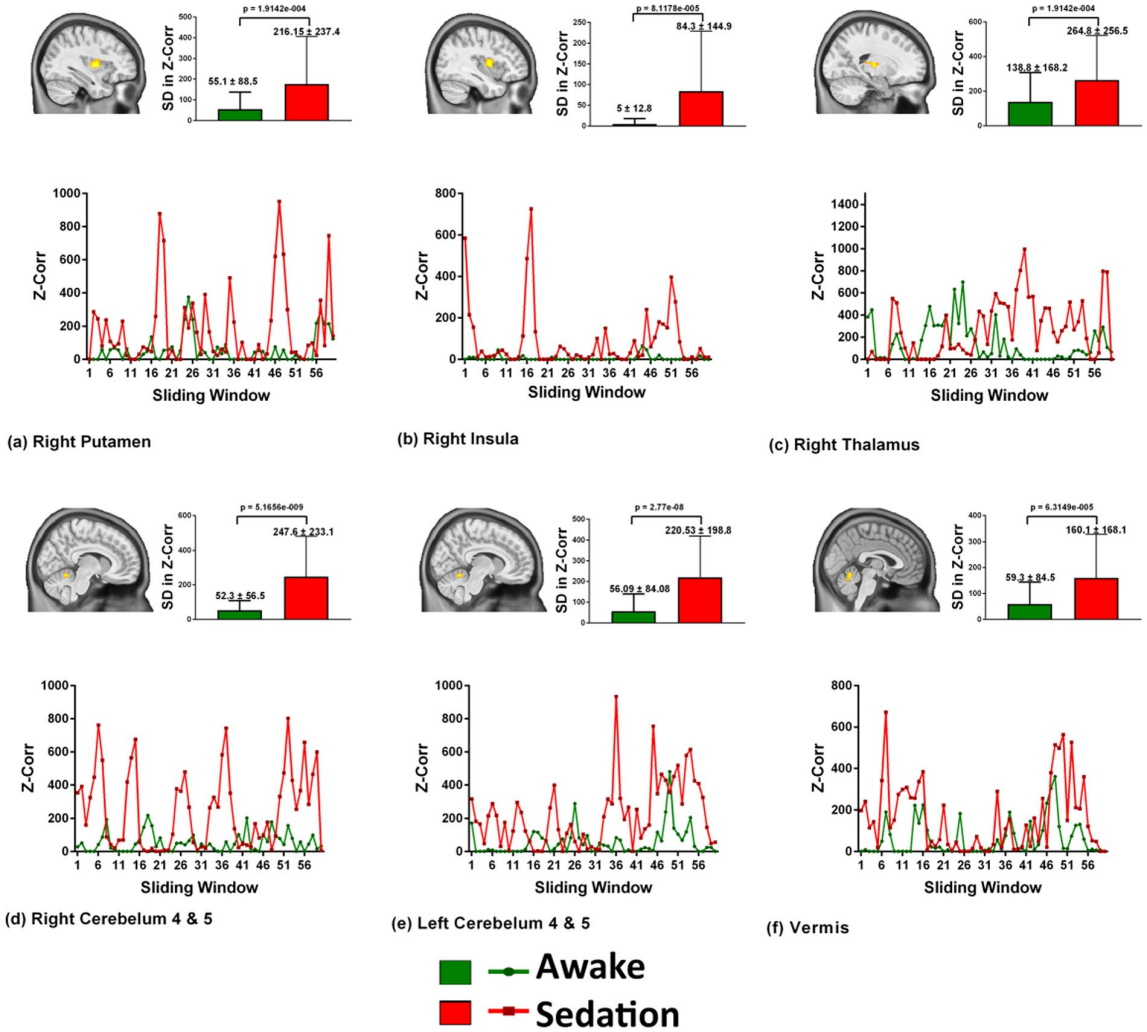
Connectivity fluctuations. Six representative deep nuclear and cerebellar regions which revealed an increased dynamic functional connectivity (DLC) during sedation is presented. The strength of the Z correlation on the Y-axis is plotted across the sliding window time points from 1 to 59 on the X-axis. The DLC during baseline awake state is depicted in green and during the sedated state is shown in red. The bar graph in the inset reveals the standard deviations (SD) in the Z correlation strengths during the two conditions. Significant sedation-induced increased connectivity involving the thalamus, basal ganglia and cerebellum with an increased SD demonstrating a “pulsatile” pattern with intermittent periods touching the zero line is most prominent in the cerebellum, basal ganglia and insula.

To visualize the temporal connectivity fluctuations in the regions that revealed significant sedation-induced differences, the mean and standard deviation of the beta values obtained from each sliding window were plotted and are depicted in Figs 3 and 4. As shown in Fig. 3, the cortical regions revealed a significant reduction in the standard deviation of the connectivity between sliding windows, i.e. connectivity fluctuations. Some areas, such as the right angular, post- and pre-central gyrus, demonstrated an almost straight-line trend with hardly any fluctuations during sedation. The pre-cuneus, bilateral supplementary motor cortex and left middle temporal gyrus had reduced connectivity with some fluctuations, but notably, these fluctuations never touched the zero line.

In contrast, the deep-seated regions (Fig. 4) revealed an increase in the connectivity strength, which is unlike the pattern observed in the cortical regions. This pattern had transient periods of increased connectivity intermixed with time points of very low connectivity, which touched the zero line at frequent intervals. This pattern was most clearly observed in the right cerebellum and putamen.

### Synchrony of the fluctuations

**Figure 5:**
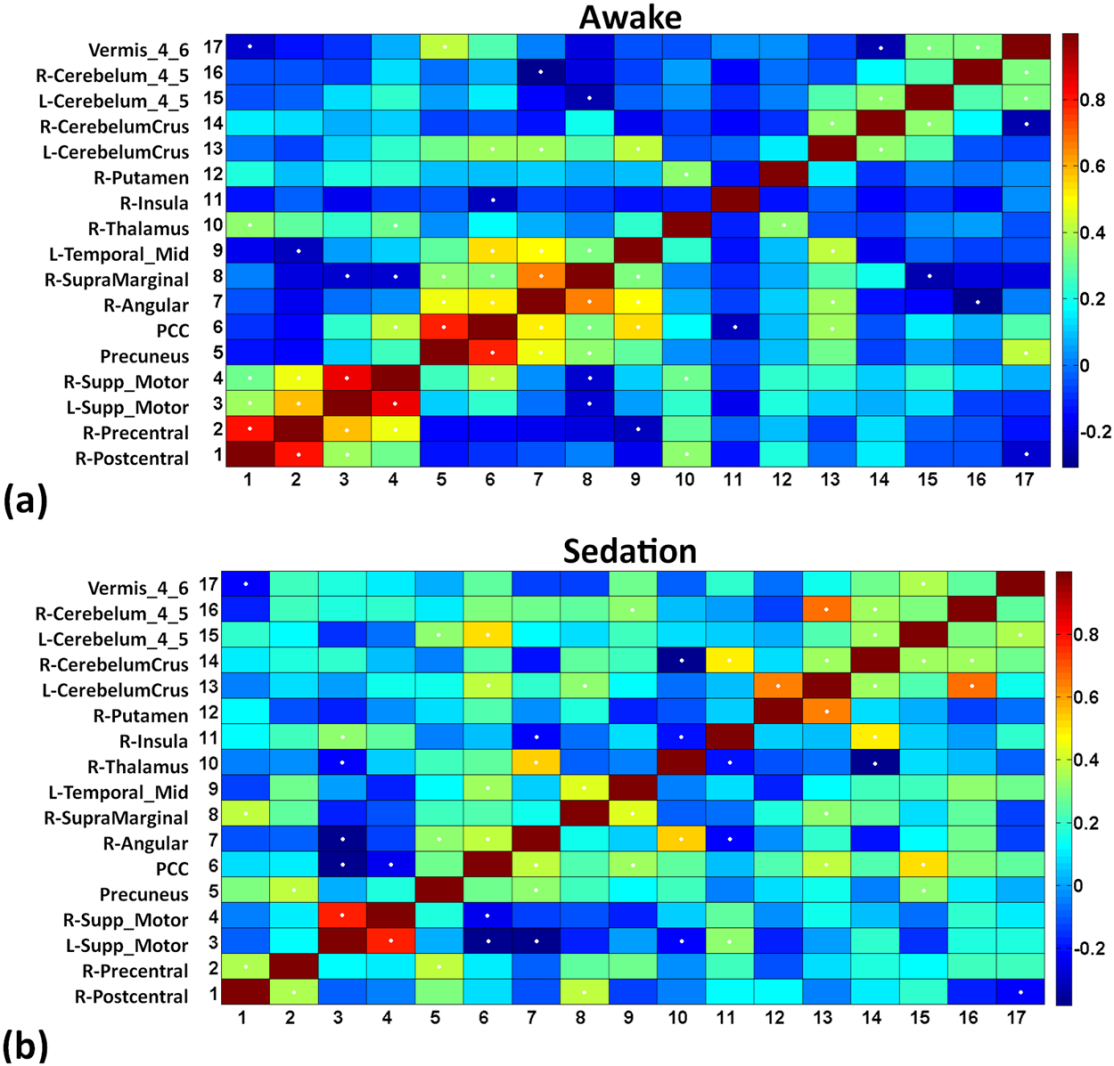
Correlation of the connectivity of the 17 areas which revealed significant changes during sedation. Baseline state (**a**) reveals significant (white stars) correlation of connection between right pre-central, post-central, and bilateral supplementary motor cortex. During sedation (**b**) only significant connection in the peri-rolandic region was between right pre-and post-central gyrus. Similar changes are also appreciable in the subcomponents of DMN (precuneus, posterior cingulate cortex (PCC), angular gyrus, and left mid-temporal region). Significantly increased connectivity of the bilateral cerebellum, vermis, and of thalamus and PCC during sedation (**b**) probably indicates re-organisation of these regions.

Since there were two distinct patterns in the DLC fluctuations visually, we were interested in determining whether these fluctuations could be interlinked. Therefore, we performed a Pearson’s correlation analysis of the DLC connectivity between the abovementioned areas (Fig. 5). We found that there was a significant positive correlation between the anatomical homologues within the DMN and around the perirolandic motor region at baseline (Fig. 5a). During sedation, the correlations among these regions were reduced. However, the connections within the cerebellar homologues that had fewer correlations at baseline had increased connectivity correlations during sedation (Fig. 5b). Several regions in the cerebellum and thalamus had increased correlations with several components of the DMN during sedation. These results indicate that during sedation, there appears to be a decreased connectivity correlation in homologous regions of the DMN and perirolandic area, and concurrently, there are increased correlations in the cerebellum and from the cerebellum and thalamus to the DMN.

### Multiband functional connectivity analysis

**Figure 6:**
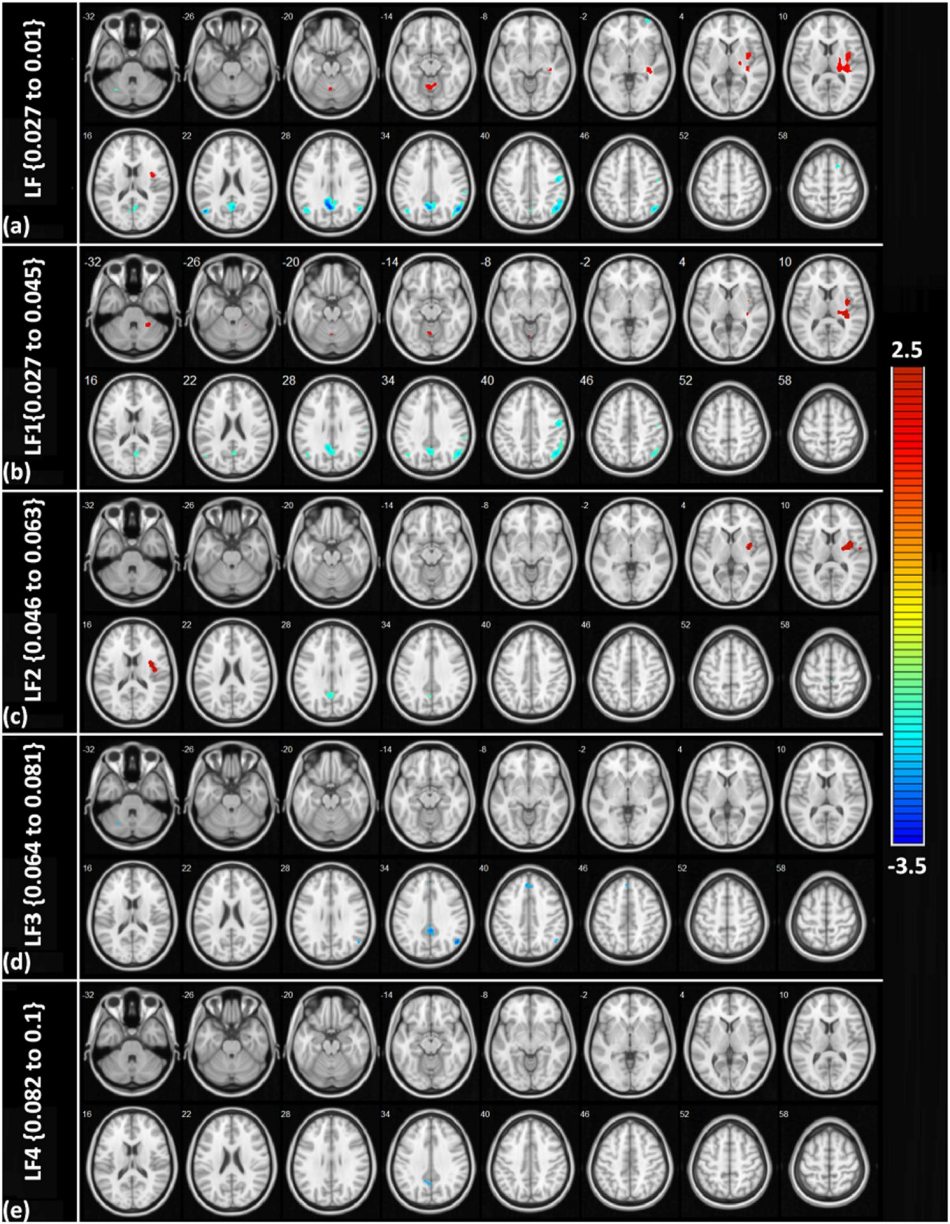
Multiband functional connectivity analysis. Rows “a to e” represent the images of the regions which showed significant differences in the DLC between baseline and sedated states. (**a**) represents the entire frequency range {0.027to 1 Hz}, (**b**) is LF1{0.027 to 0.045 Hz}, (**c**) is LF2 {0.046 to 0.063 Hz} (**d**) is LF3 {0.064 to 0.081 Hz} and (**e**) is LF4 {0.082 to 1 Hz}. It is evident that the deep nuclear regions depicted as red is only seen in rows b and c indicating a frequency range between 0.027 to 0.063 Hz.

We subdivided the entire low frequency (LF) range into four equal but arbitrary divisions (LF1{0.027 to 0.045 Hz}, LF2 {0.046 to 0.063 Hz}, LF3 {0.064 to 0.081 Hz} and LF4 {0.082 to 0.1 Hz}) and reanalysed the differences in the DLC in each frequency bands because the functional connectivity between and within different regions of the brain could be frequency-specific^31, 32^. We used bandpass filtering in the CONN toolbox to derive these groups^31^ because we were particularly interested in characterizing the contrasting changes in the temporal connectivity fluctuation patterns in the cortical and deep nuclear regions. We found that the deep nuclear regions had increased fluctuations only in the LF1 and LF2 range between 0.027 to 0.063 Hz, whereas the cortical connectivity was more widespread and was observed across the entire frequency range (0.02 to 0.1 Hz). The regions that showed significant differences across the frequency bands are depicted in Fig. 6.

## Discussion

We evaluated DLC during a baseline awake state and a propofol-induced sedation state to investigate sedation-induced changes in brain connectivity dynamics. The findings of our study have added a new dimension to the understanding of sedation-induced altered consciousness by demonstrating decreased cortical connectivity fluctuations, fewer within region correlations, and increased deep nuclear and cerebellar connectivity fluctuations, supporting the concept of a dynamic and integrated cortico-thalamic target in consciousness^14, 16^.

First, as expected, we found that several cortical regions in the DMN (PCC, pre-cuneus, angular and left middle temporal gyrus), perirolandic regions (right pre- and post-central and bilateral supplementary motor cortex), supra marginal, right middle frontal gyrus, and crus 1 and 2 of the left cerebellum had decreased cortical connectivity fluctuations during sedation. Since the average OAAS in our study was between 3 and 2, the patients were in a deeper stage of sedation and, thus, the current data are consistent with other studies that have used static connectivity measures and found decreased cortical connectivity in these regions in lower stages of consciousness^5, 9, 33–35^.

Second, we also report increased cerebellar and deep nuclear connectivity fluctuations during sedation. An increased deep nuclear and thalamic connectivity^1, 2, 9, 35^ has been reported in previous studies that included these regions in their analysis despite the differences in analysis methods, type of drugs used and levels of sedation. Previous independent reports of the loss of within and between-network integration in frontoparietal areas^8^ and thalamo-cortical anticorrelations^35^ during sedation support our findings, although those studies lacked a comprehensive evaluation of deep nuclear regions and the cerebellum. The results of the current study partially addresses these areas, and we demonstrate increased correlations in the cerebellum and between the cerebellum and thalamus to the DMN in the background of disrupted cortical networks. The findings from the current study are also consistent with the theory of an integrated cortico-cortical and cortico-thalamic complex, which is well connected within itself to maintain consciousness^16, 36^. According to this theory, consciousness is defined as our ability to assess information wholly without breaking it into pieces, such as enjoying a musical experience without breaking it into tone, pitch, temper and amplitude, which requires the integration of several cortical and subcortical regions.

The occurrence of deep nuclear connectivity in the LF1 and LF2 (0.02 to 0.06 Hz), which are below the frequency of neocortical connectivity (0.02 to 0.10 Hz), during sedation is converse to findings in the awake state. Few studies that have considered the frequency specificity of the BOLD connectivity have reported a lower frequency range of 0.01 to 0.04 Hz in the neocortical regions and a wider frequency range in the striatum during the resting awake state^37–39^. This regional frequency specificity has been ascribed to the underlying cytoarchitecture, and a wider frequency range is related to the transmodal function of the striatum, while a narrower and lower frequency range reflects the unimodal architecture of the neocortex^40^. A functional zone classification^41^ divides the brain into unimodal areas that are cytoarchitecturally well differentiated, receiving and sending information to primary sensory and motor areas, and transmodal areas that are less differentiated with diverse functions and connectivity. In light of these studies, the observations in the current study, together with previously observed evidence of an increased between-region integration, is probably indicative of a sedation-induced frequency shift in the transmodal function of the deep nuclear structures to a simpler unimodal function.

The increased correlation of the deep nuclear fluctuations within themselves and to the DMN likely to support a unimodal function, is consistent with the concept of quasi-independent modules of consciousness in the basal ganglia and cerebellum^36^. It is, however, difficult to determine from the current results whether the changes found in the deep nuclear regions, including the thalamus, are the cause of the loss of consciousness or an indirect effect associated with the loss of cortical control. Because there is evidence of cortical slowing preceding the subcortical pattern in electrophysiological measures^42^, it is likely that the deep nuclear connectivity changes occur after a certain level of loss of cortical integration. Because the role of the thalamus, cerebellum and deep nuclear structures in the loss and recovery of consciousness is well established in disorders of consciousness^43^, it is very likely that the increased fluctuations in these deep nuclear structures are important factors, and a DLC analysis of multistage studies in awake, sedated, unconscious and recovered conditions could shed more light regarding their importance.

DC has some limitations, such as a temporal resolution that is dependent on the chosen time scales and frequencies. The choice of the window width and frequency is a crucial factor that determines the results. Currently, there are no clear guidelines regarding the best choice for these variables^15, 30, 44^. We measured “dynamic local connectivity” (DLC) using voxel-wise ILC because we were primarily interested in the local connectivity based on theoretical models of cortical integration and cortical edge of instability. This method is unlike global connectivity measures and it is possible that the information derived from DLC during sedation is different than dynamic global connectivity^18^. Future studies should explore how sedation influences the interplay between DLC and dynamic global connectivity. Although we excluded frequency ranges below 0.27 Hz and above 0.10 Hz to regress the cardio-respiratory variables^30, 45^ the use of data driven methods, such as temporal ICA, for cardiac and respiratory time courses^46^ would have strengthened our conclusion, particularly since spurious frequencies at approximately 0.03 Hz have been previously associated with changes in respiratory depth^47^. It must also be noted that this study was performed with individuals with chronic low backache, which is unlike the earlier studies that involved healthy volunteers. Eight of the fourteen patients had visual analogue scale (VAS) scores ≥5 (moderate to severe pain) when they arrived at the MRI suite, necessitating the administration of fentanyl (1–1.5 µg/kg) to facilitate cooperation for the baseline awake image acquisition. This resulted in a mean VAS score of ≤4.7 ± 1.1 (mild pain) before the baseline MRI acquisition. Since it is possible that the observed alterations, particularly the deep nuclear fluctuations, could be due to pain relief during sedation, we correlated the reduction in the VAS with the mean changes in the connectivity fluctuations (Supplementary Table 1). We found no significant correlation between the pain scores and the alterations in the connectivity fluctuations. The OAAS in this study was only intermittently assessed between image acquisitions. Future studies should use simultaneous EEG-fMRI acquisitions and plasma concentrations of propofol to continuously monitor the depth of sedation during the imaging. This information, if correlated with the dynamic connectivity changes, will likely provide a more precise understanding of the stages of anaesthesia. Despite these limitations, the findings of our study have remarkably united the two theoretical models of consciousness^14, 16^, supporting the possibility that consciousness is influenced by the integrated dynamics of several cortical and subcortical networks.

## Conclusion

The current study used dynamic local connectivity resting state fMRI to demonstrate reduced cortical connectivity fluctuations and increased deep nuclear and cerebellar connectivity fluctuations during propofol-induced loss of consciousness. These findings reinforce the hypothesis that an integrated, dynamic cortical and subcortical network is a feature of consciousness.

## Materials and Methods

### Participant Selection

Fourteen subjects with chronic low backache were recruited after obtaining written informed consent. These patients were scheduled for imaging under anaesthesia because they could not lie supine in the gantry for more than 10 minutes. The experimental protocol was performed according to the required guidelines and regulations and was approved by the National Institute of Mental Health and Neurosciences (NIMHANS) Ethics Committee for conducting research on human subjects (Item No.VI, Sl.No.8.01, Clinical Neurosciences, dated 21-10-2011).

### Pre-anaesthetic evaluation and monitoring during the procedure

Individuals with a history of an allergy to propofol or eggs (due to the presence of egg lecithin in propofol), systolic blood pressure <100 mmHg, significant cardio-respiratory morbidity, potential airway difficulties, and ferromagnetic implants were excluded from the study. Individuals were also excluded if any structural brain pathology was detected on MRI. All individuals fasted for 8 hours before the propofol administration. Cardio-respiratory monitoring was performed throughout the procedure and included an electrocardiogram, non-invasive blood pressure (BP), peripheral oxygen saturation (SpO2), end-tidal carbon-dioxide (ETCO_2_) and respiratory rate (RR) using an MR compatible multi-parameter monitor (InVivo Corp., Orlando, FL, USA). An intravenous infusion of dextrose normal saline (5% dextrose in 0.9% NaCl) was delivered at a rate of 100 ml/h during the study period, and oxygen was provided through a facemask at 5 L/min flow.

### Anaesthesia protocol

A dedicated intravenous access was secured for the administration of propofol (Profol^®^, Claris Life sciences, Ahmedabad, India). Propofol was administered intravenously as a bolus dose of 1.5 mg/kg over 180 s, followed by a maintenance infusion of 1.5 mg/kg/h for the duration of the imaging using an MRI compatible syringe pump (B. Braun Space Station MRI^®^, Melsungen, Germany). The induction and maintenance of sedation and assessment of sedation quality were performed by a single individual (SK) to reduce inter-observer variations.

### Data Acquisition

Brain imaging was performed on a 3 Tesla MRI scanner (Philips Achieva 3.0 T TX, Philips Healthcare, DA Best, Netherlands) using a 32-channel head coil. The rsfMRI data were acquired twice, once immediately before the propofol sedation (baseline) and again after achieving the desired level (OAA 3-2) of sedation with propofol (Sedation). The fMRI sequence consisted of 192 dynamics whole-brain gradient-echo echo-planar images (EPI) for a total duration of 9.5 minutes. {[TR] = 3000 ms; [TE] = 35 ms; flip angle = 78; slice thickness = 4 mm; slice order: descending; number of slices = 23; matrix = 64*64*64 mm^3^, FOV = 230*230 mm^2^, voxel size = 3*3*4 mm^3^}. T1-weighted, structural imaging was performed for anatomical co-registration and segmentation. (TR:TE = 8.2:3.8, FOV = 256*256 mm^2^, voxel size of 1*1*1 mm^3^).

### Data Analysis

#### fMRI Pre-processing

The standard functional and structural MRI pre-processing steps included the following: first six functional images were discarded to allow for signal equilibration, followed by slice-time correction, realignment, co-registration, segmentation of the anatomical data for regressing the white matter (WM) and cerebrospinal fluid (CSF) effects and normalization to MNI152 standard space of 3 × 3 × 3 mm^3^ in SPM8 (http://www.fil.ion.ucl.ac.uk/spm/).

#### Head motion and artefact removal

The head movement was obtained from the realignment steps in SPM. There was no significant difference in the head motion between the baseline awake state and the sedated state (P-value of right:forward:up:: 0.8:0.6:0.6; pitch:roll:yaw:: 0.7:0.7:0.9), and the measured maximum head motion was (right:forward:up::0.42 ± 0.36:1.01 ± 0.55:0.72 ± 0.54 (mm); pitch:roll:yaw::0.01 ± 0.01:0.006 ± 0.007:0.007 ± 0.005 (degree)) during baseline and (right:forward:up:: 0.3 ± 0.5:0.9 ± 0.4:0.8 ± 0.7 (mm); pitch:roll:yaw:: 0.014 ± 0.012:0.008 ± 0.011:0.007 ± 0.01(degree)) during sedation. Before computing the connectivity maps, the head motion and physiological source noise correction was carried out using the CompCor algorithm^48, 49^. The head motion correction was carried out for the transverse axis, rotation axis and the composite motion parameter, which contains the maximum window-to-window movement and its first order temporal derivatives in the first-level general linear model^30, 48^. For the physiological noise correction, the first five principal components of the WM and CSF time-series were considered variables of no interest. Then, the data were temporally bandpass filtered with a range of 0.027-0.1 Hz to filter the irrelevant lower frequency bands, slower than the duration of a single sliding window and higher frequency, which can include cardiac and respiratory activity^30, 45^.

#### Dynamic Functional Connectivity Analysis: The sliding-window approach

A voxel-wise (Pearson’s) ILC based sliding window dynamic functional connectivity was performed using the functional connectivity toolbox (http://www.nitrc.org/projects/conn). ILC was preferred in this study because it is known to be dependent on the local connectivity with the adjacent voxels without averaging the whole brain connectivity, is less dependent on the voxel size and is less sensitive to artefacts from respiratory and cardiac cycles^5, 50^, which are critical measures that vary during sedation. To compute the dynamic connectivity differences in the present study, the entire time course of 186 dynamics was segmented into 12 dynamic (36 s) windows. By sliding the onset of this window by 3 dynamic (9 s) steps, we derived a total of 59 overlapping windows (sliding windows) in the first level analysis. We chose the shortest possible window length to maximally capture the dynamic changes based on the recommendations of an optimal duration between 30 and 60 seconds^15, 30, 44, 51, 52^. The Fisher’s z-transformed ILC maps were computed for each sliding window, yielding a set of sliding-window beta maps^30^. A second-level random-effects analysis was performed to create two within-group sample paired t-test statistical maps to identify regions with differential connectivity between the two conditions i.e., baseline and sedated state^48^. Between-group statistical parameter maps were averaged across the sliding windows and thresholded at a whole-brain cluster-level corrected α value of 0.05 for a voxel-wise P-value of < 0.05 with a false-discovery rate (FDR) correction and a minimum cluster extent of 10 contiguous voxels. The brain regions with significant connectivity differences were visualized in multi-slice brain images using ‘xjView’ (http://www.alivelearn.net/xjview8/) and BrainNet Viewer (http://www.nitrc.org/projects/bnv/). The strength of the connectivity dynamic was estimated by multiplying the conjugate cluster (k) with the corresponding intensity (t) values at each voxel to derive the corresponding Beta values from each sliding window z-map. The fluctuations in connectivity were measured by the mean and SD of these beta values^30^. To ascertain significant differences in the connectivity fluctuations, paired two-sample t-tests with an α value of 0.05 were used along with corrections for multiple comparisons (p = 0.05/59 = 0.00084). The fluctuations in brain connectivity in the significant areas are shown in Figs 3 and 4.

## Electronic supplementary material

Supplementary Information
