# Supplementary material for: Dynamic local connectivity uncovers altered brain synchrony during propofol sedation
Source: Sci Rep. 2017 Aug 17;7:8501. doi: 10.1038/s41598-017-08135-2 (PMC5561230; doi:10.1038/s41598-017-08135-2)
Supplement: Supplementary file 1 — Supplementary Information [file 41598_2017_8135_MOESM1_ESM.doc]

**Dynamic functional connectivity uncovers altered brain synchrony during propofol sedation**

Rose Dawn Bharath^1^, Rajanikant Panda^1^, Jitender Saini^1^, Kamath Sriganesh^2,*^, G.S. Umamaheswara Rao^2^

^1^Department of Neuro Imaging and Interventional Radiology (NIIR), ^2^Department of Neuroanaesthesia, National Institute of Mental Health and Neuroscience (NIMHANS), Bangalore, India

*Corresponding author: cns.researchers@gmail.com

| **Brain Region** | **Corelation Val** | **P-Value** |
| --- | --- | --- |
| Precuneus | 0.169233358 | 0.59 |
| PCC | -0.050641534 | 0.87 |
| Right Angular | -0.071345333 | 0.82 |
| Right Supra Marginal | 0.031228167 | 0.92 |
| Right Post-central | -0.086937651 | 0.78 |
| Right Pre-central | -0.148614728 | 0.64 |
| Left Supplementary Motor | 0.117690092 | 0.71 |
| Right Supplementary Motor | 0.168836462 | 0.59 |
| Right Middle Frontal | -0.180227544 | 0.57 |
| Left Middle Temporal | 0.328922099 | 0.29 |
| Right Putamen | -0.211990581 | 0.5 |
| Right Insula | -0.162928211 | 0.62 |
| Right Thalamus | 0.155624818 | 0.66 |
| BL Cerebellum_4_5 | 0.138428462 | 0.66 |
| Vermis_4_5 | 0.497664333 | 0.09 |
| Left Cerebellum Crus 1 & 2 | -0.440065935 | 0.15 |

**Supplementary Table 1:** Correlation of connectivity with reduction in visual analog pain scores (VAS). The strength of connectivity in the areas which revealed significant changes after sedation found no significant correlations with reduction of VAS after IV Fentanyl.
